# Supplementary material for: Domain-interface dynamics of CFTR revealed by stabilizing nanobodies
Source: Nat Commun. 2019 Jun 14;10:2636. doi: 10.1038/s41467-019-10714-y (PMC6572788; doi:10.1038/s41467-019-10714-y)
Supplement: Supplementary file 1 — Supplementary Information [file 41467_2019_10714_MOESM1_ESM.pdf]

	FR1	CDR1	FR2	CDR2	FR3	CDR3	FR4
D12	QVQLQESGGGLVQAGSSLRLACAAT	GSIRSINN	MGWYRQAPGKQRGMVAI	ITRVGNTD	YADSVKGRFTISRDNAKNTVYLQMNSLKPEDTATYYC	HAEITEQSRPFYLTDD	YWGQGTQVTVSSAAA
T2a	QVQLQESGGGLVQAGGSLRLSCAAS	GSIFRIDA	MGWYRQAPGKQRELVAH	STSGGSTD	YADSVKGRFTISRDNAKNTVYLQMNSLKPEDTAVYYC	NADVRTRWYASNN	YWGQGTQVTVSSAAA
T27	QVQLQESGGGLEQPGGSLRLSCATS	GVIFGINA	MGWYRQAPGKQRELVAT	FTSGGSTN	YADFVEGRFTISRDNAKNTVYLQMNGLRPEDTAVYYC	HATVVVSRYGLTYD	YWGQGTQVTVSSAAA
T4	QVQLQESGGGLVQAGGSLRLSCAAS	GSTFAIIA	MGWYRQAPGKQRELVAV	ISTGDTR	YADSVKGRFTISRDNAKNTVYLQMDSLRPEDTAVYYC	NAAVQVRDYRN	YWGQGTQVTVSSAAA
T8	QVQLQESGGGLVQPGGSLRLSCAAS	GSTSSINA	MGWYRQAPGKQREPVAI	SSSGGDTR	YAEPVKGRFTISRDNAQNKVYLQMNSLKPEDTAVYYC	WLNWGRTSVN	SWGQGTQVTVSSAAA
G3a	QVQLQESGGGLVQAGGSLRLSCTAS	GRAFSWYV	MGWFRQAPGKEREFVAT	VSGNGSRRD	YADSVKGRFTISRDNAKNTVYLQMNSLKPEDTAVYYC	AASSTYYYTDPEKYD	YWGQGTQVTVSSAAA

Supplementary Figure 1. Multiple alignment of the selected nanobody sequences. Amino acid sequences of D12, T2a, T27, T4, T8 and G3a nanobodies selected for this study. The complementarity-determining region (CDR) sequences alternating with framework (FR) sequences were identified according to International ImMunoGeneTics information system amino acid numbering (http:// http://www.imgt.org/). The alignment has been generated using ClustalX.

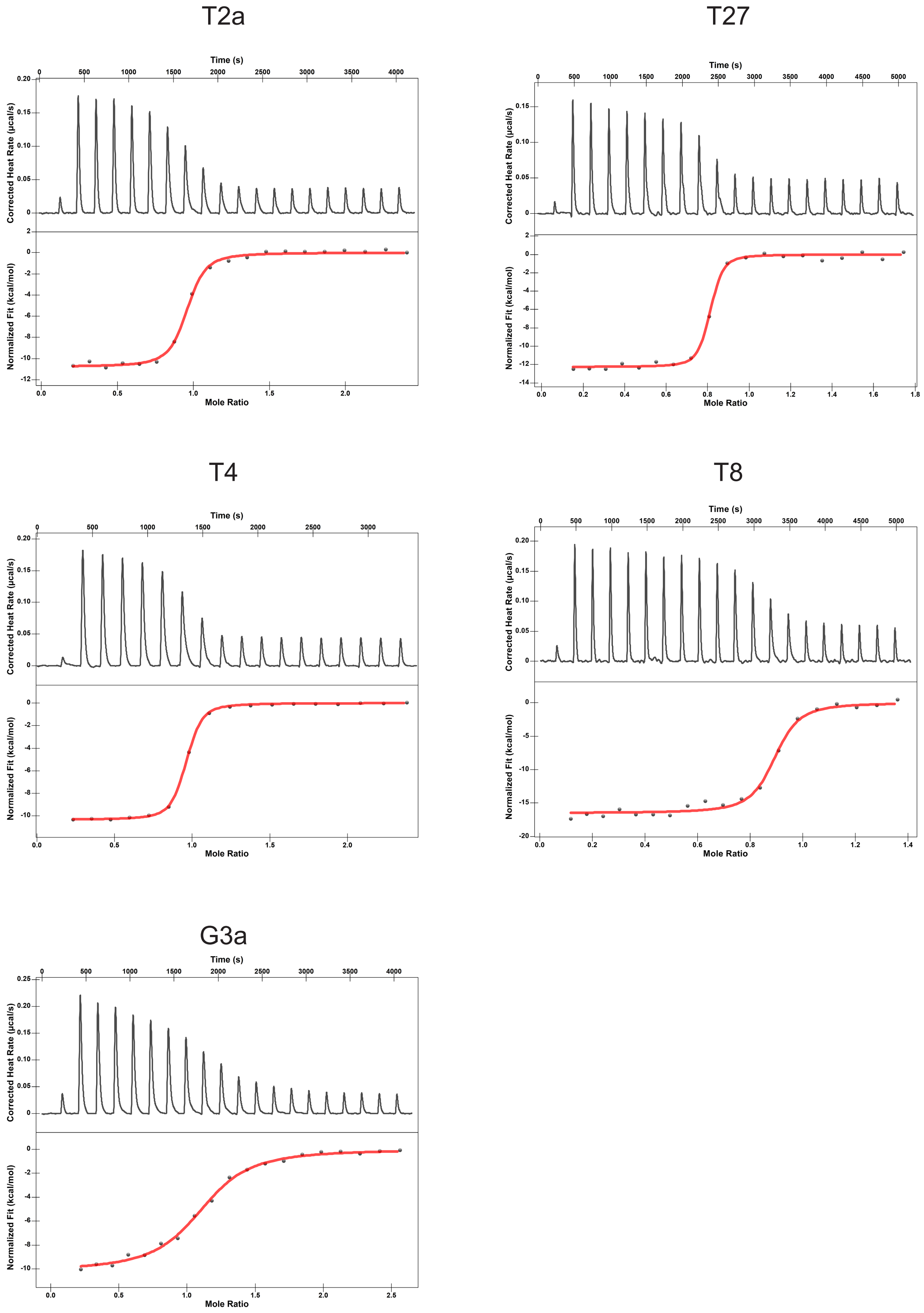

Supplementary Figure 2 | Representative thermograms obtained by titrations of nanobodies T2a, T27, T4, T8 and G3a into 2PT-NBD1 at 20°C.
Upper panels show raw data, and lower panels represent the integration of heat changes associated with each injection of nanobodies. Data were fitted using a one-site binding model as described in Methods. Computated parameters are presented in Figure 1. Representative curve of 3 independent experiments is shown.

a

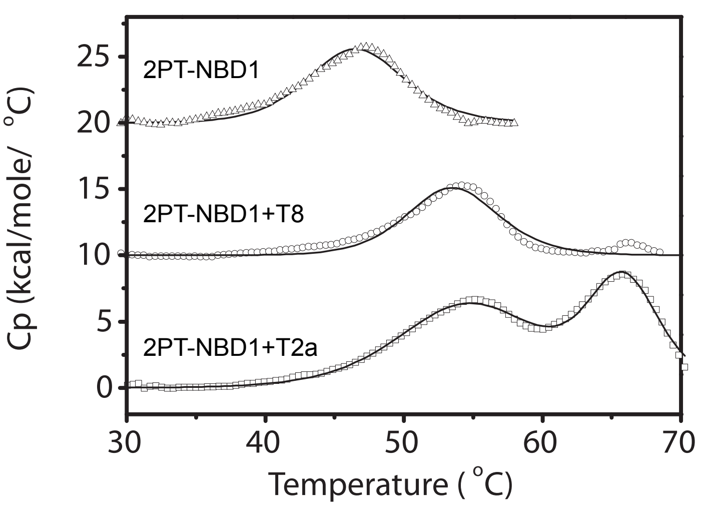

b

Sypro 2.5x	Tm (°C)	Sypro 2.5x	Tm (°C)
2PT-NBD1	44.2 ± 0.5	F508del-2PT-NBD1	41.3 ± 0.4
+ T2a	55.3 ± 0.5	+ T2a	52.4 ± 0.2
+T27	55.6 ± 0.7	+T27	53.4 ± 0.3
+D12	58.5 ± 0.3	+D12	54.4 ± 0.2
+T4	56.6 ± 0.2	+T4	40.3 ± 0.6
+T8	53.5 ± 0.3	+T8	40.7 ± 0.3
+G3a	42.8 ± 0.6	+G3a	41.5 ± 0.7
Sypro 2.5x	Tm (°C)	Sypro 5x	Tm (°C)
T2a	66.1 ± 0.7	T2a	65.5 ± 1.5
T4	71.9 ± 0.5	T4	71 ± 1.0
T8	nd	T8	65.8 ± 0.2
T27	63.7 ± 0.2	T27	63.3 ± 0.3
D12	65.2 ± 0.1	D12	68.8 ± 0.9
G3a	nd	G3a	78 ± 1.0

c

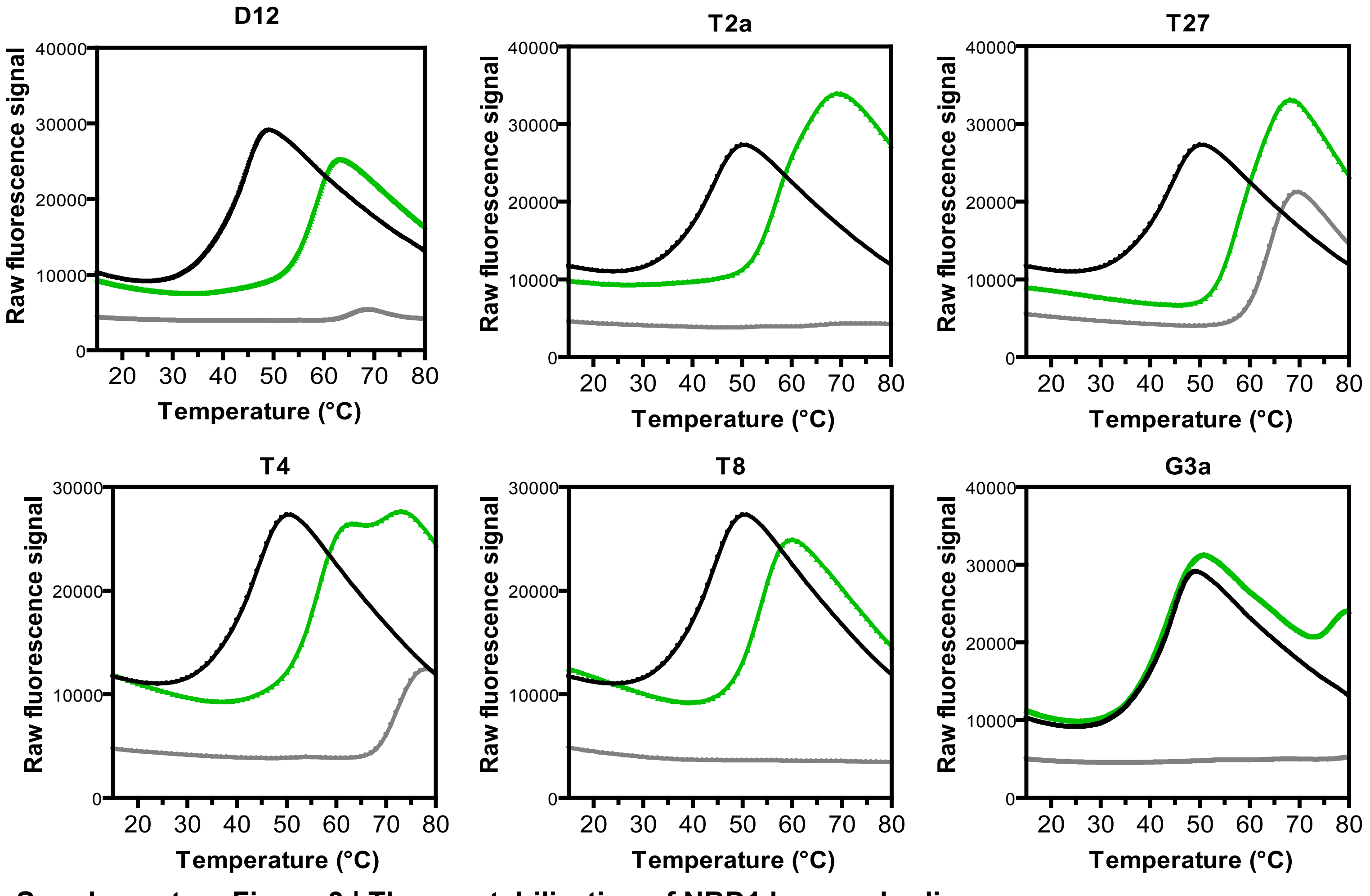

Supplementary Figure 3 | Thermostabilization of NBD1 by nanobodies.
(a) Stacked overlay of DSC fitted curves obtained with 2PT-NBD1 alone or stabilized with nanobodies T2a and T8. Representative curve of 2 independent experiments is shown. (b) Summary table of melting temperatures of 2PT-NBD1 and F508del-2PT-NBD1 in absence and/or presence of different nanobodies (top panel) determined using DSF as in panel (c). The lower part of the table shows melting temperatures of isolated nanobodies at 2 different concentration of Sypro-Orange dye. Data are mean ± SEM of duplicates from four independent experiments. (c) Raw fluorescence signal of thermal unfolding scans of 2PT-NBD1 in the absence (black curves) and presence of nanobody (green curves) were acquired by DSF using 2.5x Sypro-Orange. Unfolding of nanobody alone is depicted as grey curves. Curves depict mean of duplicates of one experiment representative of three independent experiments.

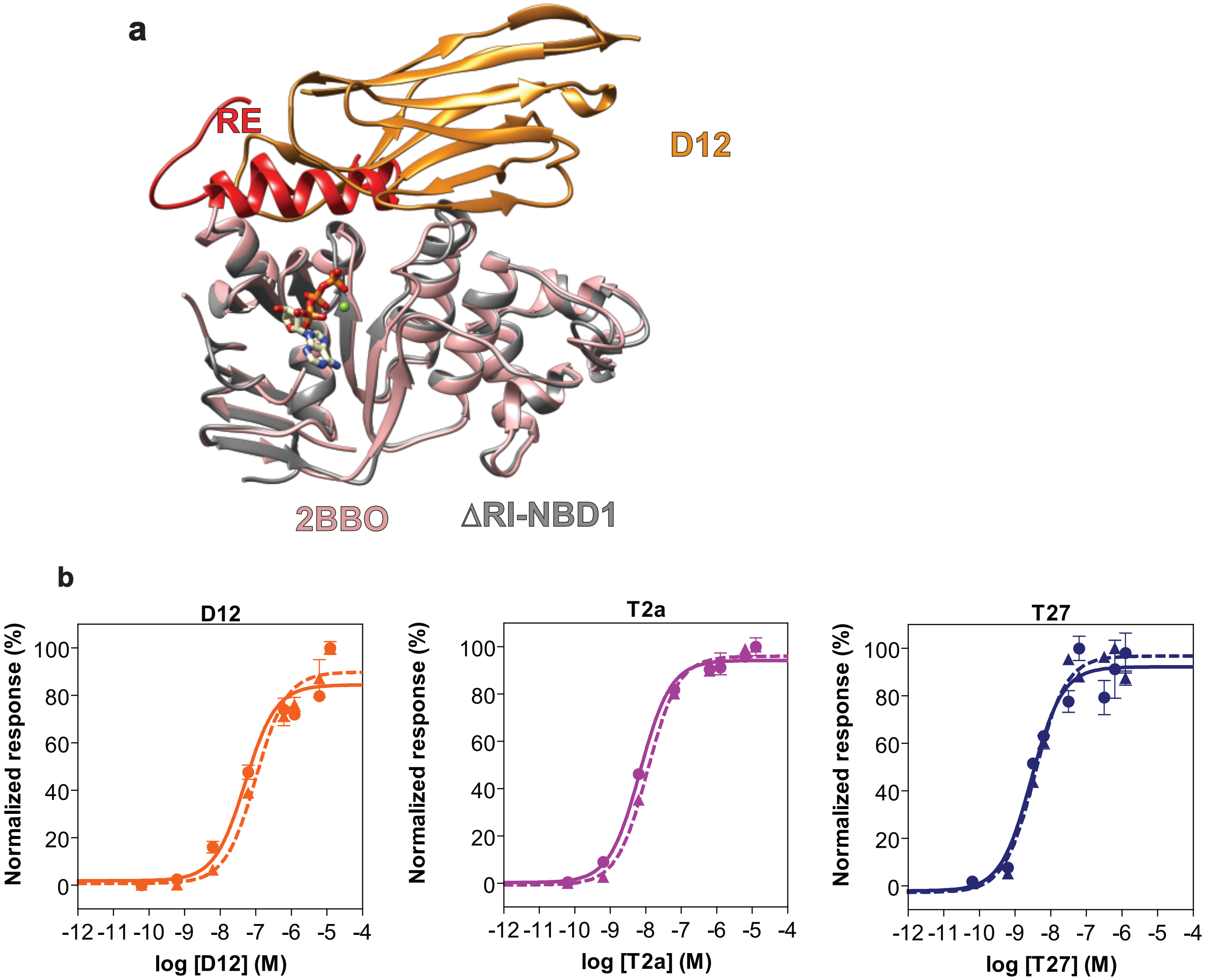

Supplementary Figure 4 | Regulatory Extension (RE) of NBD1 does not impede binding of D12, T2a and T27 nanobodies

(a) Superimposition of published structure of human NBD1 (PDB: 2BBO) and the structure of ΔRI-NBD1 in complex with nanobody D12, showing overlap between the nanobody and the RE. (b-c-d) Dose-response ELISA showing nanobodies D12, T2a, T27 binding to 2PT-NBD1-RE (dashed lines) or 2PT-NBD1 (solid lines), as described in Figure 1a. Representative curve of 3 independent experiments is shown. Error bars represent the standard deviation (SD) of duplicates. Data were normalized to maximum signal for each nanobody separately.

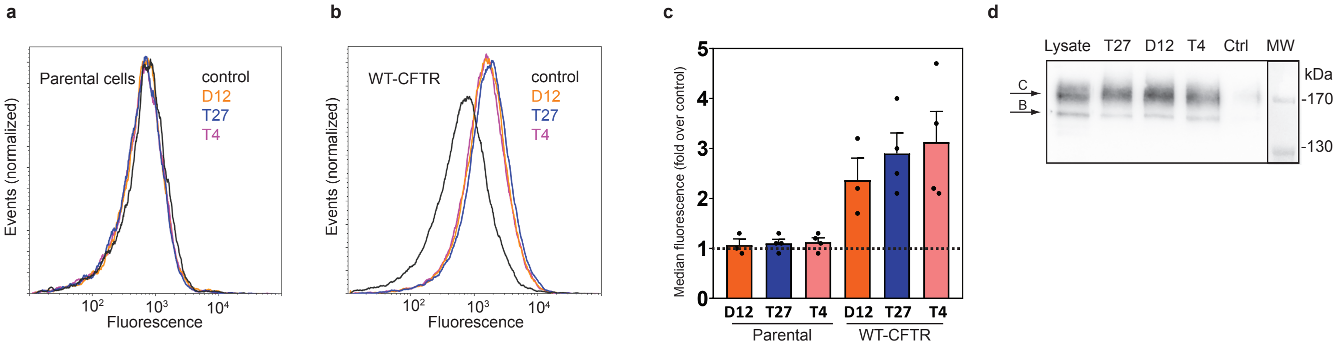

Supplementary Figure 5

(a) Flow cytometry analysis of nanobodies D12, T27 and T4 on parental BHK-21 cells show no difference in labelling compared to a negative control nanobody while in (b) increased labelling is observed for the NBD1-specific nanobodies in BHK-21 cells overexpressing wt-CFTR. Data were normalized to the number of events acquired in each condition. Graph depicts one representative of at least three independent experiments. (c) Average median fluorescence (fold over negative control) for each of the three nanobodies as illustrated in panel (a) and (b). Average of at least 3 independent experiments (± SEM). (d) Immunoblot of CFTR from solubilized BHK-21 cells pulled-down with His-tagged nanobodies. Eluted nanobodies-CFTR complexes were separated by SDS-PAGE and presence of CFTR was detected with 596 antibody after immunoblotting. Arrows indicate the mature (band C) and immature (band B) forms of CFTR.

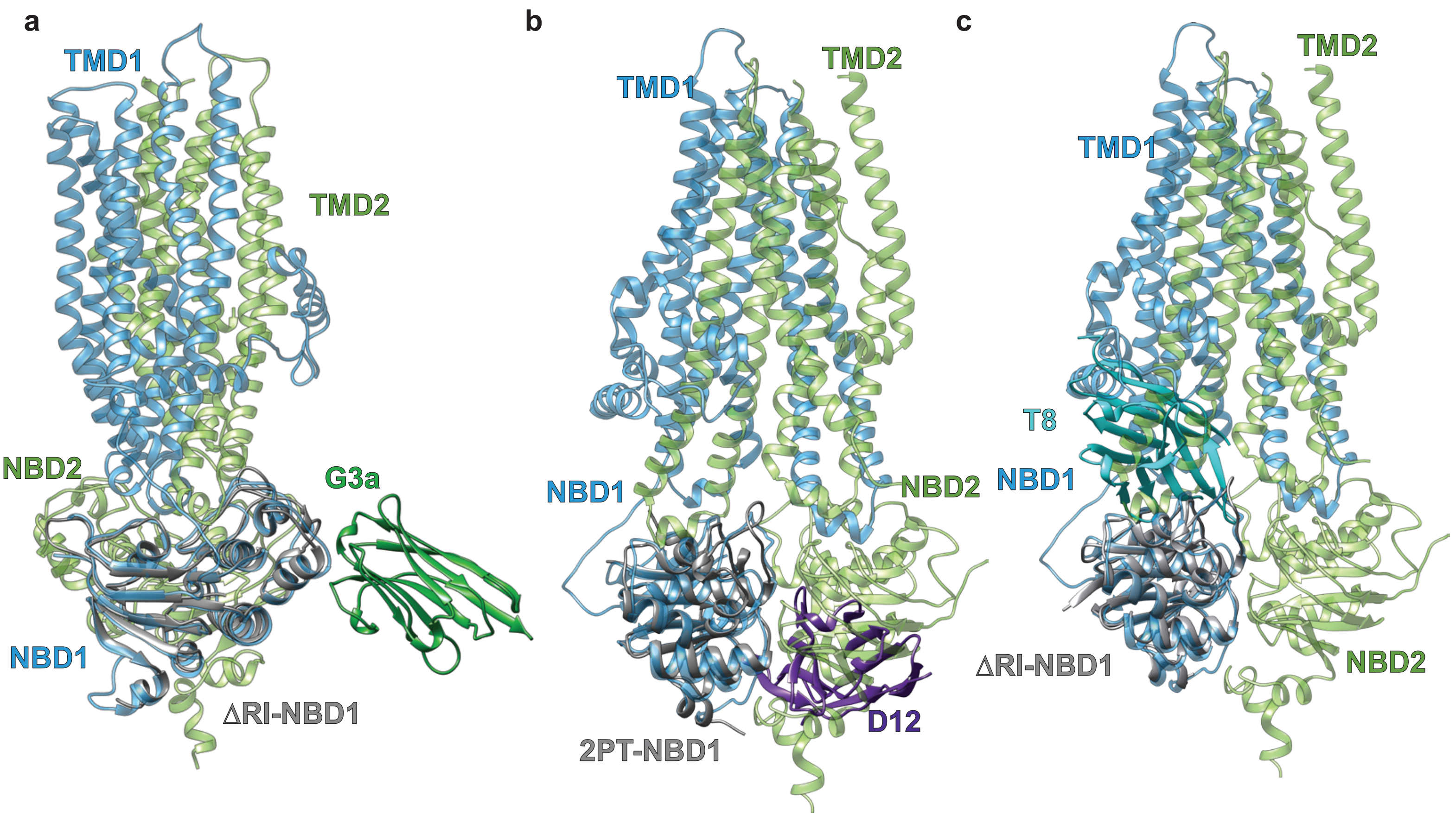

Supplementary Figure 6 | NBD1-nanobody complexes superimposed onto phosphorylated CFTR.

(a) Superimposition of the structure of zebrafish CFTR structure (PDB: 5W81) and ΔRI-NBD1 in complex with nanobody G3a structure showing that binding of G3a is also compatible with the phosphorylated state of CFTR. (b) Superposition of the 2PT-NBD1:T2a complex with the same CFTR structure shows that T2a binding is incompatible with the closing of the NBD1 observed in the ATP-bound CFTR structure. (c) Same superimposition as in (a) with the structure of ΔRI-NBD1 in complex with nanobody T8 suggesting a different conformational state for which a large motion of NBD1 is necessary to permit T8 binding.
